# The implication of neutrophil extracellular traps in nonalcoholic fatty liver disease

**DOI:** 10.3389/fimmu.2023.1292679

**Published:** 2023-11-02

**Authors:** Pengyan Fa, Benjamin G. Ke, Abigail Dupre, Allan Tsung, Hongji Zhang

**Affiliations:** ^1^ Department of Surgery, School of Medicine, University of Virginia, Charlottesville, VA, United States; ^2^ School of Medicine, University of Virginia, Charlottesville, VA, United States

**Keywords:** nonalcoholic fatty liver disease (NAFLD), neutrophil extracellular traps (NETs), immune response, liver inflammation, pathophysiological progression

## Abstract

Nonalcoholic fatty liver disease (NAFLD) is an expanding worldwide health concern, and the underlying mechanisms contributing to its progression still need further exploration. Neutrophil extracellular traps (NETs) are intricate formations comprised of nuclear constituents and diverse antimicrobial granules that are released into the extracellular milieu by activated neutrophils upon various triggers, which play a pivotal part in the onset and advancement of NAFLD. NETs actively participate in the genesis of NAFLD by fostering oxidative stress and inflammation, ultimately resulting in hepatic fat accumulation and the escalation of liver injury. Recent insights into the interaction with other hepatic immune populations and mediators, such as macrophages and T regulatory cells, have revealed several important mechanisms that can trigger further liver injury. In conclusion, the formation of NETs emerged as an important factor in the development of NAFLD, offering a promising target for innovative therapeutic approaches against this debilitating condition. This comprehensive review seeks to compile existing studies exploring the involvement of NETs in the genesis of NAFLD and their influence on the immune response throughout the progression of NAFLD.

## 1 Introduction

NAFLD is the prevailing chronic liver ailment, distinguished by the excessive buildup of lipids in the liver. The prevalence of NAFLD is on the rise primarily due to increasing rates of obesity and metabolic syndrome (1). NAFLD activity score (NAS) and Non-invasive scoring systems (NSS) are designed for clinical use to identify and evaluate NAFLD progression (2–4). Progression stages have been broadly recognized and are derived from simple fatty liver disease (steatosis) without specific hepatocellular inflammation (5). NAFLD can advance into severe forms such as nonalcoholic steatohepatitis (NASH), cirrhosis, and hepatocellular carcinoma (HCC) due to a diverse array of factors, encompassing lipotoxicity-induced endoplasmic reticulum (ER) stress and mitochondrial dysfunction (6, 7), activated Kupffer cells (KCs) (8), immune cell-mediated inflammatory processes (9, 10), and gut microbiota (11–13).

Recent studies have highlighted the significant role of gut microbiota in NAFLD. Whole-genome shotgun (WGS) sequencing performed by Loomba et al. revealed that levels of *Escherichia coli* and *Bacteroides vulgatus (B. vulgatus)* were increased in patients with advanced fibrosis, while *Eubacterium rectale* and *B. vulgatus* were increased in patients with mild/moderated NAFLD (14). Mouries, et al. further found an initial disruption of the intestinal epithelial barrier and gut vascular barrier (GVB) in NASH (15). In recent years, NETs stimulation by microorganisms such as adherent-invasive *Escherichia coli* (AIEC) (16) and *Entamoeba histolytica (E. histolytica)* (17) has been observed. One recent study found aberrant intestinal neutrophil migration, increased bacterial translocation in the circulation, and higher lipopolysaccharides (LPS) level in the visceral adipose tissue (VAT) in interleukin-17 (IL-17) receptor-deficient (IL-17RA-/-) mice fed with a HFD (18). These findings collectively suggest that gut microbiota may influence the NETs in NAFLD. Nevertheless, additional investigation is needed to directly confirm the effect of gut microbiota on NETs in the development of NAFLD.

Over the past two decades, there has been an increasing emphasis on investigating the influence of immune cells on the development of NAFLD towards NASH-fibrosis. In the early 2000s, studies began to highlight the importance of inflammatory processes in the progression of NAFLD. For example, in an article published in the journal Gastroenterology in 2002, Sanyal, A. J. et al. showed that individuals diagnosed with NASH exhibited elevated liver inflammation levels compared to those with steatosis (19). Since then, numerous studies have investigated the involvement of diverse immune cells and inflammatory agents in the onset and advancement of NAFLD, and some investigations have demonstrated the implication of macrophages, T cells, and cytokines in the pathogenesis of hepatic inflammation and fibrosis during NAFLD (20–25).

Neutrophils, a subset of leukocytes, serve as part of the primary line of defense in the immune system, tasked with protecting the body from infections and illnesses by engaging in the destruction of pathogens like viruses, bacteria, and fungi (26–28) through phagocytosis, degranulation, and NETosis (29, 30). Brinkmann, V. et al. first proposed the term “NETs” in 2004, marking the beginning of a new era (31). NETs are reticulated extracellular formations consisting of chromatin, granular proteins, and histones. During NAFLD, hepatic lipid accumulation can prompt an inflammatory reaction, inducing neutrophil activation and subsequent NETs release. NETs can initiate additional inflammation and attract other immune cells to the liver, such as macrophages and T regulatory cells (Treg), ultimately contributing to NASH-HCC development (32, 33). In the context of these studies, our research team observed that NETs foster inflammation and facilitate the progression of hepatocellular carcinoma in NASH, providing a novel strategy that targets NETs for chronic liver disease therapy. Within this comprehensive review, we begin by consolidating the research regarding the involvement of NETs at various phases of NAFLD advancement, especially their interaction with the immune microenvironment during NAFLD progression. Finally, we conclude by discussing the potential therapeutic approaches targeting NETs to fight NAFLD.

## 2 NETs in immune defense

NETs released by activated neutrophils were first reported in 2004 as a physical barrier that helps trap and degrade virulent pathogens and kill bacteria. Deoxyribonuclease1 (DNase1) treatment abolished NETs formation, which is consistent with the observation that NETs are primarily composed of DNA (31). In the subsequent year, the same research team discovered that granular proteins but not histones facilitated the destruction of both yeast-form and hyphal cells of *Candida albicans* in the antimicrobial action of NETs (34). As a foremost innate immune responder to inflammation and tissue injury, neutrophils are considered crucial in bolstering immune surveillance. The synergy between NETs and neutrophil elastase (NE), histones, or other constituents enhances the efficacy of antimicrobial capabilities. This immune defense process is called “NETosis.” Along with eliminating bacteria, NETs also contribute to fighting viruses and fungi (35–40).

NETs are involved in anti-inflammatory functions and have been shown to be relevant in trapping and killing *Staphylococcus aureus* (41). In 2014, Schauer C. et al. reported that aggregated NETs limit chronic inflammation by degrading cytokines and chemokines through binding with proteases (42). In 2019, Ribon M. et al. proposed that NETs exert anti-inflammatory actions in rheumatoid arthritis via complement component 1q (C1q) and human cationic antibacterial protein (LL-37) (43).

The liver is the most critical organ responsible for maintaining normal host homeostasis. During sepsis-related organ injury, it relies on various cell types, including KCs, hepatocytes, B cells, and neutrophils, to carry out pivotal functions in combating bacterial infections (44, 45). The bacteria are captured and removed by resident KCs, which are localized in the liver sinusoids (46, 47). Subsequently, neutrophils migrate and accumulate in the infected area, where they interact with platelets and release neutrophil extracellular traps to capture and clear bacteria (48).

**Figure 1:**
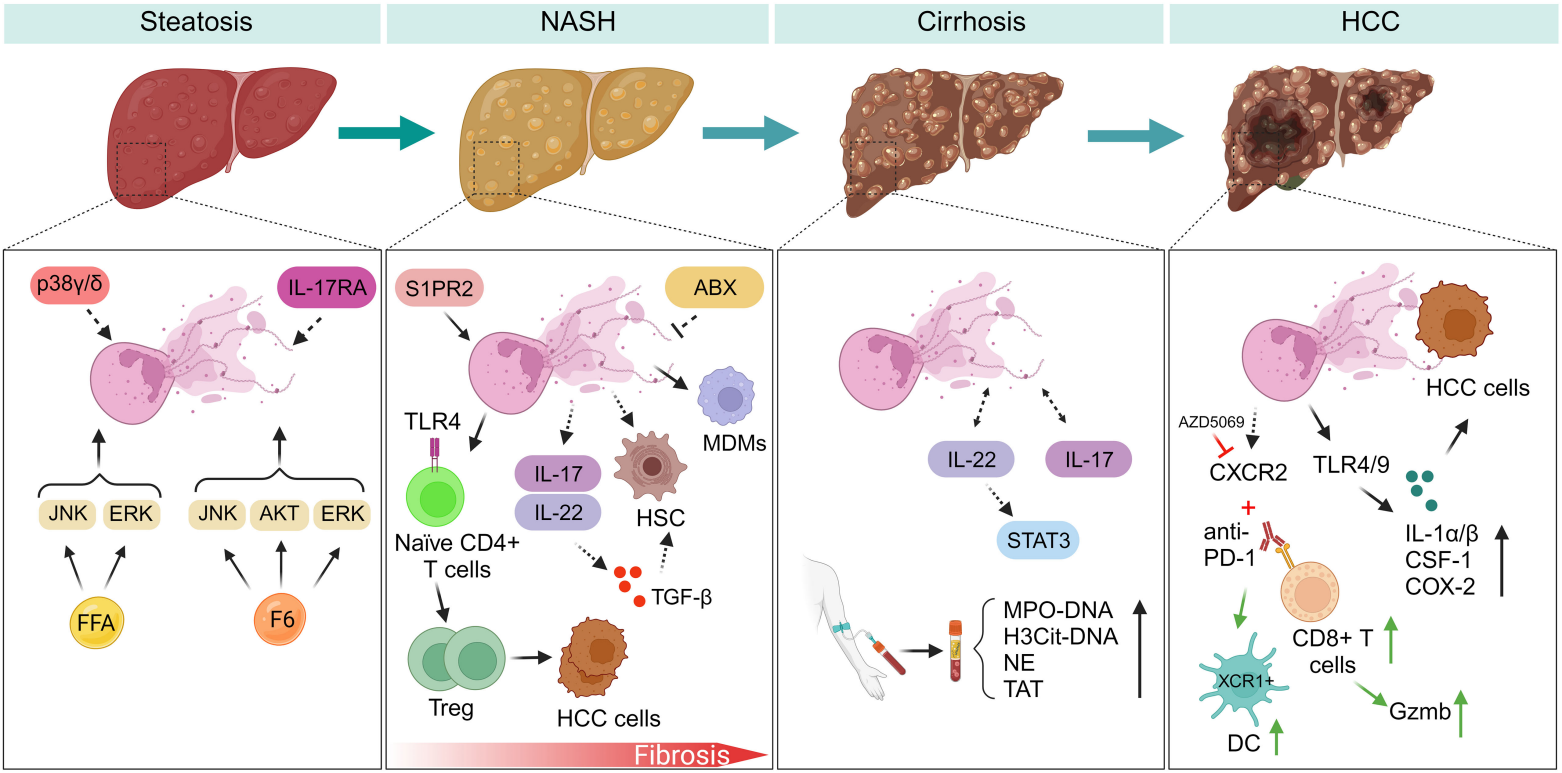
NETs regulate inflammatory response during NAFLD progression. (Steatosis) Myeloid cells with p38γ/δ deficiency are resistant to HFD-induced steatosis. F6 induces NETs formation through activating ERK, JNK, and AKT signaling pathways. However, FFAs induce NETs formation by activating ERK, JNK, but not AKT kinase. IL-17RA-/- mice fed with HFD have experienced decreased intestinal neutrophil migration, indicating gut microbiota may be a potential modulator of NETs formation during NAFLD. (NASH-fibrosis) In the progression of NASH-fibrosis, S1PR2 functions as a catalyst for NETs formation, and silencing S1PR2 can mitigate hepatic fibrosis and inflammation. NETs generation fosters the differentiation of Treg and facilitates the advancement of HCC. NETs entice MDMs, subsequently reshaping the inflammatory milieu within NASH. Neutrophils activate HSC by inducing the production of ROS and MPO. ABX treatment may inhibit NETs formation during liver fibrosis. IL-17 and IL-22 produced by neutrophils promote liver fibrosis development through TGF-β signaling. (Cirrhosis) NETs markers exhibit a marked increase in patients with liver cirrhosis and portal vein thrombosis. During liver cirrhosis, NETs formation assumes a role in promoting coagulation. IL-22 and IL-17 production from neutrophils potentially promote NETs formation through STAT3 signaling during NAFLD. (HCC) Neutrophils expressing CXCR2 infiltrate in the course of NASH-HCC development, and a combined therapy involving PD-1 antibodies and CXCR2 inhibitor (AZD5069) reshapes the behavior of TANs. HCC cells internalize NETs, leading to elevated COX2 levels through activation of TLR4/9. FFA, free fatty acid; F6, furanoid F-acid F6; ERK, extracellular signal-regulated kinase; JNK, c-Jun N-terminal kinase; AKT, protein kinase B; IL-17RA, interleukin-17 (IL-17) receptor; S1PR2, specific G protein-coupled S1P receptor 2; ABX, antibiotics; IL-17, interleukin-17; IL-22, interleukin-22. STAT3, signal transducer and activator of transcription 3; MDMs, monocyte-derived macrophages; TAT, thrombin-antithrombin complex; PVT, portal vein thrombosis; HCC, hepatocellular carcinoma; ROS, reactive oxygen species; MPO, myeloperoxidase; CSF-1, colony stimulating factor 1; XCR1, X-C motif chemokine receptor 1; DC, dendritic cells; PD-1, programmed death protein-1; CXCR2, CXC chemokine receptor 2; TANs, tumor-associated neutrophils; COX2, cyclooxygenase-2; IL-1a/β, interleukin-1a/β.

Excessively expressed NETs have been observed to contribute to inflammation within the liver (49–51). In this review, we will discuss how NETs regulate inflammatory response during NAFLD progression (**Figure 1**). Prior research has revealed that NETs fulfill a dual function, participating in both pro- and anti-inflammatory processes. Hence, it becomes imperative to comprehend their formation and function under both typical and pathological circumstances to devise precise therapeutic approaches for NAFLD.

## 3 NETs in the pathophysiological progression of NAFLD

As of July 2023, there are over 5000 publications in the PubMed database that pertain to the exploration and comprehension of the various roles of NETs. Since 2018, the study of the potential significance of NETs in the progression of NAFLD has been steadily gaining prominence (**Table 1**). The collective discoveries across various stages of NAFLD suggest that NETs actively contribute to pro-inflammatory processes, hastening the advancement of the disease.

**Table 1:** The involvement of NETs in the development of NAFLD.

Year	Immune responses		Phenotype	Study model
	Innate	Adaptive		
2018 (32)	Neutrophils infiltration ↑ Macrophage infiltration ↑	N/A	• MPO ↑ • H3Cit ↑ • Tumor formation ↑	STAM mouse model
2019 (52)	Neutrophils infiltration ↑	• CD4+ T cells ↓ • CD8+ T cells ↓ • T cell exhaustion ↑	• H3Cit-DNA ↑ • Tumor formation ↑	STAM mouse model
2020 (53)	Neutrophils infiltration ↑	N/A	• MPO ↑ • H3Cit ↑ • S1P ↑ • NETosis ↑ • Neutrophil spontaneous apoptosis ↓ • Liver fibrosis ↑	MCD-HFD mouse model
2021 (33)	Neutrophils infiltration ↑ Macrophage infiltration ↑ DC ↑	• CD4+ T cells ↓ • Treg ↑ • B cells ↑	• H3Cit ↑ • OXPHOS ↑ • Tumor formation ↑	STAM and WD mouse models
2021 (54)	N/A	N/A	• MPO ↑ • H3Cit ↑ • TAT ↑	HCC and cirrhosis patients
2022 (55)	N/A	N/A	• MPO ↑ • H3Cit ↑ • NE ↑ • TAT ↑	Liver cirrhosis patients with PVT
2022 (56)	N/A	N/A	• TAT ↑ • CT ↓ • Coagulation ↓ • Thrombin and fibrin formation ↑	NASH patients
2022 (57)	N/A	N/A	• MPO ↑ • H3Cit ↑ • NE ↑ • Platelet ↑ • NETs/IL-1β/IL-17A colocalization ↑	NASH and hepatitis patients
2023 (58)	Neutrophil infiltration ↑ Macrophage infiltration ↑	• CD4+ T cells ↓ • CD8+ T cells ↑	• MPO ↑ • H3Cit ↑ • NE ↑	MCD-HFD and HFHC-NASH mouse models

N/A, data not available; MPO-DNA, myeloperoxidase associated DNA; H3Cit-DNA, histone H3 associated DNA; S1P, sphingosine 1-phosphate; MCDHF, methionine/choline deficient diet-high fat diet; DC, dendritic cells; OXPHOS, oxidative phosphorylation; WD, western diet; STAM, stelic animal model, HCC, hepatocellular carcinoma; NE, neutrophil elastase; CT, coagulation time; TAT, thrombin-antithrombin; PVT, portal vein thrombosis; IL-6, interleukin-6; IL-1β, interleukin-1β; HFHC, high fat and high cholesterol diet.

### 3.1 NETs in steatosis

As a central part of the innate immune response, neutrophil infiltration in the liver has a notable role in promoting NAFLD progression. Myeloid cells lacking p38 mitogen-activated kinases p38γ and p38δ (p38γ/δ) demonstrate resistance to high-fat diet-induced steatosis in association with reduced neutrophil infiltration in the liver. Conversely, wild-type mice with excessive neutrophil infiltration experience enhanced steatosis development (59). The generation of NETs is one of the critical strategies of neutrophils during an inflammatory response; however, whether NETs participate in the development of steatosis is yet to be fully explored.

Peptidylarginine deiminase 4 (PAD4) is essential for the formation of NETs, as PAD4^-/-^ neutrophils lose the ability to form NETs (60). Two constituents of the DNase1 family, DNase1 and DNase1 like 3 (DNase1L3), have been identified as contributors to NETs formation both *in vitro* and *in vivo* (61). Aberrant lipid accumulation resulting in lipotoxicity is considered to be a crucial event in hepatic steatosis progression. Elevated production of free fatty acid (FFA) represents a significant hallmark of NAFLD (62, 63). Inhibition of fatty acid synthase (FASN) in human primary liver microtissues prevents the development of steatosis (64). Our research has demonstrated that free fatty acids (FFAs), such as linoleic acid (LA) and palmitic acid (PA) but not oleic acid (OA), induce the formation of NETs *in vitro.* However, inhibiting NETs through DNase1 or using PAD4 knockout mice did not prevent the increase in FFAs, which suggests that NETs formation is not a causative factor of steatosis but rather an outcome of lipid accumulation (32). Nevertheless, the mechanism under this circumstance still requires further exploration. By employing gas chromatography–mass spectrometry (GC-MS) to examine peripheral blood from individuals, researchers discovered that F6 (furanoid F-acid F6) instigates NETs formation through activating ERK (extracellular signal-regulated kinase), JNK (c-Jun N-terminal kinase), and AKT (protein kinase B) kinases. On the other hand, other common fatty acids such as PA, palmitoleic acid (PO), stearic acid, and OA induce NETs formation by activating ERK, JNK, but not AKT kinase (65). Additionally, in response to lLPS) stimulation, neutrophils release NETs via toll-like receptor 4 (TLR4)-JNK axis activation (66). Thus, it is possible that steatosis induces NETs formation through these distinct pathways.

### 3.2 NETs in NASH

NASH represents a progressed stage of NAFLD, displaying a robust correlation with both inflammation and metabolic disturbances. Neutrophil infiltration in NASH was identified decades ago (67). NETs, one of the key features of neutrophils, have emerged as modulators of chronic inflammation and subsequently promote the progression of cancers (68–70). In 2018, our research team initially documented the occurrence of NETs formation in NASH, noting elevated levels of myeloperoxidase (MPO)-associated DNA (MPO-DNA), a hallmark of NETs, in the serum of preoperative NASH patients. To investigate further, we utilized STAM mice, a NASH mouse model created by neonatal streptozotocin (STZ) injection followed by a high-fat diet (HFD) regimen (71). In this established NASH model, we found that NETs formation was accompanied by increased neutrophil infiltration and inflammatory cytokines. NETs regulate the inflammatory environment in NASH by recruiting monocyte-derived macrophages (MDMs). Our study provides fundamental evidence that the formation of NETs is a vital factor in driving the advancement of NASH, bridging the gap between steatosis and NASH.

Additionally, our research team discovered that NETs serve as a link between adaptive and innate immunity via promotion of regulatory T cell differentiation and function (33). In this investigation, a murine model was subjected to a western diet to induce a NASH phenotype, unveiling a direct relationship between heightened Treg activity and the generation of NETs. Moreover, the inhibition of Treg led to the prevention of NASH liver development. This fascinating discovery is dependent on the mitochondrial oxidative phosphorylation (OXPHOS) pathway in naïve CD4 positive T cells with TLR4 mediating metabolic reprogramming. NETs exhibit a vital role in the induction of hypercoagulability in NASH patients. In this study, plasma samples obtained from both NASH patients and healthy donors underwent assessment, and NETs isolated from NASH patients showed a higher level of procoagulant activity and pro-inflammatory factors than those from healthy controls (56). A recent publication revealed that changes in linoleic acid and γ-linolenic acid (GLA) are responsible for initiating the formation of NETs in an early NASH mouse model, suggesting that fatty acids play a crucial role in regulating NETs formation in the context of NASH (58).

### 3.3 NETs in NASH-fibrosis

Hepatic fibrosis plays a critical role in determining the mortality rates of NASH patients and is also linked to the long-term prognoses of individuals diagnosed with NAFLD (72, 73). A prominent factor during NASH-fibrosis development is hepatic stellate cell (HSC) activation (74, 75). The interaction between neutrophils and HSC in liver fibrosis has been studied for years. For example, increased infiltration of neutrophil-derived IL-17A exhibits advanced liver fibrosis through promoting HSC activation (76, 77). Neutrophils activate HSCs through reactive oxygen species (ROS) and MPO production (78, 79). However, our understanding of the role of NETs during liver fibrosis is still limited, and the role of NETs in modulating the interaction between neutrophils and HSC is a promising direction for future research.

MPO serves as a central element and inflammatory enzyme within neutrophils. MPO-deficient neutrophils led to an inability to form NETs, underscoring the essential role of MPO in NETs formation (80). Within the NASH-fibrosis experimental framework triggered by a high-fat diet lacking methionine and choline, the deficiency of MPO in knockout mice led to a notable decline in fibrosis. This finding implies a potential contribution of NETs to the advancement of NASH-fibrosis (81).

Specific G protein-coupled S1P receptor 2 (S1PR2) acts as a stimulator of Sphingosine 1-phosphate (S1P), another key responder of inflammation. A recent study shows that knockdown of SIPR2 can decrease liver inflammation and fibrosis by inhibiting NETs formation (53). The presence of S1PR1 has been identified as essential for the recruitment of neutrophils (82). Considerable research has been carried out on the involvement of S1P receptors in both adaptive and innate immunity and its influence on various immune cell types, such as T cells, B cells, NK cells, macrophages, dendritic cells, and neutrophils (83–88). Although few studies directly explain the immune response involving NETs and liver fibrosis, the impact of NETs on immune responses could be one of the reasons for the progression of liver fibrosis.

### 3.4 NETs in cirrhosis

Liver cirrhosis is the irreversible end stage of chronic fatty liver disease. The pathophysiologic diagnosis of liver cirrhosis is characterized by hepatocellular dedifferentiation, fibrous scarring, HSC activation, and increased collagen deposition (89, 90). Cirrhosis can be classified into two clinical stages: compensated and decompensated, also known as asymptomatic and symptomatic stages. According to the U.S. Department of Veterans Affairs, patients with compensated cirrhosis with conditions such as HCC or advanced decompensated cirrhosis are considered eligible for liver transplantation.

The increased prevalence of bacterial infection in individuals diagnosed with cirrhosis has been established for over three decades (91–93). Early studies have shown that liver cirrhosis patients have compromised neutrophil recruitment, resulting in impaired immune response (94, 95). Changes in neutrophils, such as impaired NADPH oxidase activity and reduced MPO release, could explain the increased vulnerability to bacterial infections in individuals with decompensated cirrhosis (96). Interleukin-22 (IL-22) facilitates the development of cirrhosis to HCC through Signal transducer and activator of transcription 3 (STAT3) signal activation (97). Neutrophils have been shown to be one of the sources for IL-22 production (98), and IL-22 secreted from T cells can recruit neutrophils to peripheral tissues (99). Moreover, IL-22 and IL-17 production from neutrophils promote NETs formation (100, 101). The association between NETs and IL-22 during NAFLD is unclear. New investigations have been conducted to elucidate the involvement of NETs in liver cirrhosis. Markers of NETs formation, MPO-DNA and citrullinated histone H3 (H3Cit) associated DNA (H3Cit-DNA), were significantly elevated in patients with cirrhotic livers compared to healthy controls (54). Additionally, in cases of liver cirrhosis with portal vein thrombosis (PVT), NETs have been shown to enhance procoagulant activity (55). Elevated NETs levels in cirrhotic livers with PVT may act as a link to malignancy in HCC (102).

### 3.5 NETs in NASH-HCC

The role of NETs in HCC, the most common form of primary liver cancer, is becoming increasingly recognized. Our previous research has indicated that the invasion of neutrophils and the creation of NETs play a role in the progression of HCC within the context of NASH (32). We observed macrophage infiltration at eight weeks in the STAM NASH mouse model, whereas neutrophil infiltration was observed at five weeks. Notably, the number of KCs decreased at an early age, potentially being replaced by infiltrating macrophages. This observation aligns with recent studies reporting a decrease in KCs during NASH, subsequently replaced by infiltrating lipid-associated macrophages (LAMs) (9, 103, 104). Collectively, these reports suggest that NETs may interact with macrophages and contribute to the development of NASH-HCC. However, the association between NETs and other immune cells requires further investigation.

Cancer cells induce NETs formation, stimulating cancer cell invasion and migration (105, 106) through activation of various pathways (107, 108). NETs-DNA promotes cancer metastasis by interacting with the protein coiled-coil domain containing 25 (CCDC25) (109). In the NASH-HCC mouse model, there was an observed reduction in CD4+ and CD8+ T cells, accompanied by elevated levels of PD-L1 and indicators of T cell exhaustion (52). Furthermore, NETs interact with regulatory T cells (Treg) and promote their activity by modulating the metabolic reprogramming of naive CD4+ T cells. Depletion of Treg has been shown to prevent the development of HCC in NASH (33), further demonstrating a potential mechanism by which NETs in NAFLD interact with other immune cells. Additionally, the internalization of NETs by HCC cells increased COX2 expression through Toll-like receptors TLR4 and TLR9 activation (110).

Research has indicated a correlation between heightened oxidative stress and inflammation with the formation of NETs in individuals suffering from liver cirrhosis and HCC. The buildup of NETs in the liver can trigger liver injury and foster fibrosis, ultimately leading to cirrhosis and elevating the risk of HCC. A recent study shows that CXCR2-positive neutrophils infiltrate NASH-HCC models, and anti-PD-1/CXCR2 inhibitor combination therapy reprograms tumor-associated neutrophils (TANs). However, it still needs to be examined whether or not NETs participate in this process (111).

## 4 Potential therapeutic targeting of NETs in NAFLD

Therapeutic targeting of NETs in NAFLD is an active area of research. NETs have been implicated in the transformation of NAFLD to its more severe form, NASH, and have been shown to be associated with liver fibrosis, cirrhosis, and HCC. Several potential therapeutic strategies are being explored for targeting NETs in NAFLD, including:

### 4.1 Anti-inflammatory agents

In a clinical investigation, asthma patients undergoing daily treatment with inhaled corticosteroids (ICS) displayed notably reduced mean plasma NETs level compared to patients who either did not use ICS or only used them infrequently (112). Studies have revealed that the antioxidant drug resveratrol (RESV) can effectively reduce the generation of NETs by neutrophils in individuals afflicted with severe COVID-19 infection (113). This finding reveals a potential role for RESV in mitigating the development and accumulation of NETs in the liver. During a preclinical investigation, the combination of DNase1 with hydroxychloroquine (HCQ) or aspirin, a nonsteroidal anti-inflammatory drug (NSAID), exhibited a remarkable inhibition of HCC metastasis in a murine model (110). Moreover, in a rat model with hepatic fibrosis elicited by thioacetamide (TAA) administration, the experimental groups treated with low-dose aspirin, high-dose aspirin, and enoxaparin exhibited a significantly reduced liver fibrosis score when compared to the untreated group (114).

### 4.2 NETs-degrading enzymes

Enzymes that can break down NETs, such as DNase1, have been used for treatment of NETs formation for decades. In a mouse model of necrotizing fasciitis, Group A Streptococcus (GAS) expressing DNase (Sda1) has been identified as a contributor to bacterial virulence. Sda1 effectively breaks down NETs both *in vitro* and *in vivo* (115). Studies have demonstrated that DNase exhibits therapeutic potential in animal models of NASH-HCC (32).

### 4.3 Anticoagulants

Medications that inhibit blood clotting, including heparin and warfarin, have been found to decrease NETs formation and enhance liver function in mouse models of NAFLD (116, 117). In a rat model administered with chronic carbon tetrachloride (CCl4) to establish liver fibrosis, low molecular weight (LMW)-heparin and dalteparin sodium significantly ameliorated hepatic fibrogenesis (118). Additionally, in prothrombotic factor (F) V Leiden mutant mice, C57BL/6 wild-type mice, and warfarin-treated mice exposed to CCL4, experimental results showed that warfarin effectively reduced hydroxyproline content and fibrosis score (119). Moreover, in a cirrhotic Wistar rats model, notable reductions in liver fibrosis, HSC activation, and desmin expression were found in the enoxaparin treated group (120). Consistently, rivaroxaban (RVXB), an oral anticoagulant, also dramatically reduced HSC activation and intrahepatic microthrombosis in CCL4-induced cirrhosis rat model (121). Along with these preclinical studies, the first clinical trial was established in 2012. In this trial, 70 patients with advanced cirrhosis were randomly assigned to two groups, with or without enoxaparin treatment. No patients in the enoxaparin group had PVT (122). Although no direct evidence has shown that enoxaparin limits NETs accumulation, it provides a potential direction for further exploration of its role in NETs formation.

### 4.4 Vitamins

Vitamin C is an essential vitamin for human health. A previous study has shown that vitamin C-deficient neutrophils do not undergo NETosis (123). In this study, L-gulono-γ-lactone oxidase (Gulo)-/- mice, which are deficient in vitamin C synthesis, undergo decreased neutrophil apoptosis even without a hypoxic environment. Similar results were found in sepsis mouse models where NETs formation was dramatically decreased in vitamin C-deficient mice (124, 125). In contrast to the role of vitamin C in NETs formation, another study showed that 1, 25-dihydroxyvitamin D_3_ can induce NETs formation and the mRNA levels of NETs-related markers (126). In this study, NETs demonstrated a potential protective role during infections by sequestrating the spread of pathogens.

### 4.5 Probiotics, prebiotics and synbiotics

Numerous studies have demonstrated that an imbalance in gut microbiota can disrupt the homeostasis of the intestinal system, thereby increasing the risk of advanced NAFLD (127–132). The activation of NETs by microbiota has been reported previously (133). NETs can be stimulated in a rat model of LPS-induced sepsis, and the disruption of NETs has been shown to ameliorate intestinal injury (134). Depletion of microbiota through antibiotics (ABX, a mixture of Ampicillin, Streptomycin, Metronidazol and Vancomycin) treatment was associated with a decrease in NETs formation (135). These findings suggest that gut microbiota-targeted treatments, such as the use of PPS, hold promise as potential interventions to limit NETs formation during NAFLD (136, 137). Although PPS exhibit positive effects on gut homeostasis, their direct impact on NETs during NAFLD remains unclear. In mouse bone marrow–derived neutrophils (BMDNs) and human promyelocytic cell line HL-60, the probiotic *L. rhamnosus* strain GG (LGG) has been found to inhibit NETs formation, potentially by suppressing ROS and phagocytosis (138).

Nevertheless, these potential therapeutic strategies are currently in the preliminary stages of research, necessitating further investigations to determine their effectiveness and safety in human populations. Exploring immune system modulation to reduce the formation and accumulation of NETs and addressing the root causes of inflammation and oxidative stress may present novel therapeutic avenues for individuals with NAFLD. It is important to note that while targeting NETs in NAFLD is an important avenue of research, the management of NAFLD requires a comprehensive approach that addresses the underlying causes of oxidative stress and inflammation, such as obesity, insulin resistance, and poor dietary habits.

## 5 Future perspectives

Targeting NETs in NAFLD holds promise for improving its management and preventing progression to more severe forms like NASH and cirrhosis. Further laboratory studies and clinical trials are needed to develop new drugs that specifically target NETs formation in NAFLD. These drugs could be used alone or in combination with existing therapeutic strategies to improve liver health and reduce the risk of liver cirrhosis and HCC (139, 140). As our understanding of the role of NETs in NAFLD improves, personalized medicine approaches may gain wider acceptance. These may involve genetic and biomarker-based approaches to identify patients at high risk of developing NAFLD and tailoring therapeutic strategies accordingly (141–143). Combining multiple therapeutic strategies, such as anti-inflammatory agents (114), NET-degrading enzymes (115), anticoagulants (116), immune modulation, and vitamin C (125) may also provide synergistic benefits and enhance the efficacy of NETs-targeted therapies. To assess the effectiveness and safety of NETs-targeted therapies in human populations, extensive clinical trials on a large scale are necessary. These trials should be rigorously designed, randomized, controlled, and inclusive of participants at different stages of NAFLD (10). NETs-targeted therapies should be integrated with existing therapies for NAFLD, such as lifestyle modifications, weight loss, and medications to improve insulin sensitivity to maximize their therapeutic benefits. Overall, the future of NET-targeted therapies for NAFLD holds great promise, and further research in this area could transform the treatment of chronic liver diseases and improve the health outcomes of millions of individuals worldwide.

## 6 Conclusion

NETs play a complex role in the pathogenesis of NAFLD. While they are critical to the body’s defense against infection and inflammation, their excessive formation and accumulation can contribute to liver damage and disease progression, potentially resulting in liver failure. Additional investigations are essential to fully elucidate the precise mechanisms through which NETs impact the development of NAFLD and to identify effective strategies for targeting NETs in NAFLD. This pursuit may involve exploring new drugs, personalized medicine approaches, and combination therapies, as well as their integration with existing treatments.

To comprehensively understand the mechanism of NETs in NAFLD, it is essential also to examine their role in other common liver diseases. This broader perspective can contextualize our current knowledge of NETs in NAFLD amidst liver-related conditions. For example, a previous study demonstrated that acute alcohol consumption reduces LPS-induced NETs formation during alcohol hepatitis (AH) in mice (144). The effect may be dependent on the elevated levels of IL-6. In other sterile liver inflammation scenarios, such as ischemia/reperfusion (I/R) injury, NETs formation is induced by damage-associated molecular patterns (DAMPs), activating TLR4 and TLR9 signaling pathways (145).

Furthermore, the imbalance of the gut-liver axis (GLA) during NAFLD has been known for years (146). Previous studies have shown that microorganisms can stimulate the formation of NETs (16, 17). Therefore, gaining insights into the connection between gut microbiota and NETs in NAFLD represents a promising avenue for research. This exploration provides an opportunity to develop new microbiota-based therapies, such as PPS treatment. The potential benefits of probiotics in delaying NAFLD development through the modulation of the LPS/TLR4 signaling pathway have been examined (147). Moreover, investigations into prebiotics and synbiotics in the context of NAFLD (148, 149) suggest that combining the benefits of gut microbiota treatments with NETs inhibition may offer a novel approach to mitigate NAFLD severity.

Finally, the interplay between NETs and other immune cells in the development of NAFLD needs deeper exploration. Reports have highlighted interactions between NETs and Treg, along with elevated levels of dendritic cells (DCs) and B cells during NAFLD (33). Emerging evidence also suggests an increased infiltration of macrophages in NAFLD (32, 33, 58, 103). These ongoing investigations imply that NETs may influence a complex network within the immune environment during NAFLD. A more thorough understanding of these interactions may pave the way for innovative therapeutic strategies aimed at managing NAFLD and its complications through immune response modulation.

## Author contributions

PF: Conceptualization, Visualization, Writing – original draft, Writing – review & editing. BK: Writing – review & editing. AD: Writing – review & editing. AT: Conceptualization, Funding acquisition, Supervision, Writing – review & editing. HZ: Conceptualization, Funding acquisition, Supervision, Writing – review & editing.
